# A possible anti-inflammatory mechanism of ethyl acetate extracts of *Teucrium stocksianum* Bioss

**DOI:** 10.1186/s12906-015-0834-x

**Published:** 2015-08-29

**Authors:** Syed Muhammad Mukarram Shah

**Affiliations:** Department of Pharmacy, University of Malakand, Chakdara, Dir Pakistan

**Keywords:** *Teucrium stocksianum*, Anti-inflammatory mechanism, Histamine, Bradykinin, Arachedonic acid

## Abstract

**Background:**

*Teucrium stocksianum* (*T. stocksianum*) is one of the important members of the genus *Teucrium* which contains numerous biologically active compounds. Traditionally, it is used for the treatment of fever, pain, as expectorant and blood purifier. Researchers are trying to discover plants origin, novel and safe remedies for the management of various ailments. The present study was aimed to determine the possible anti-inflammatory mechanism of ethyl acetate extract of *T. stocksianum.*

**Methods:**

Preliminary, the ethanolic extract and sub-fractions were screened for anti-inflammatory potential at doses of 100, 200 and 300 mg/kg (i.p) body weight, using carrageenan induced paw edema test in mice. In-order to determine the possible mechanism of anti-inflammatory effect, the ethyl acetate fraction was ascertained with different phlogistic agents like histamine, bradykinin, prostaglandins E_2_ and arachedonic acid via paw edema test in mice.

**Results:**

The ethanolic extract and sub-fractions of *T. stocksianum* displayed marked to moderate anti-inflammatory activity in a carrageenan induced paw edema test in mice. Among the sub-fractions, ethyl acetate fraction (EAF) demonstrated excellent (66 %) anti-inflammatory action at the highest tested dose (300 mg/kg) that reached to the maximum value at 3^rd^ hour after carrageenan injection and remained significant (***P < 0.001) till 5^th^ hour of test sample administration. EAF revealed moderate effect against the paw edema induced by histamine (31.048 %) while non-significant results (18.148 %) were observed against the edema induced by bradykinin. The extract demonstrated significant (66.23-73.076 %) anti-inflammatory potential against the edematogenic effect of prostaglandin E_2_. Moreover, the extract also significantly inhibited (51.33 %) the paw edema induced by arachedonic acid.

**Conclusion:**

Our results suggest that the EAF has dual action and produced the anti-inflammatory effect by blocking both pathways of arachedonic acid metabolites (cyclooxygenase and lipoxygenase). Thus validating the traditional use of *T. stocksianum* and could provide a source of novel, effective and safe drug for the treatment of inflammation.

## Background

In the plant kingdom nature has bestowed the remedies of ailments. According to WHO, approximately 70-80 % population of developing countries acquire their primary pharmaceutical care from medicinal plants [1]. Medicinal plants have diversified pharmacological potentials due to the presence of various classis of phytochemicals [2].

Lamiaceae is a famous cosmopolitan family comprising of about 200 genera and more than 4000 species disseminated all over the world particularly in the Mediterranean and mountainous region [3]. Phytochemicals evaluation of the family Lamiaceae revealed the presence of different classes of secondary metabolites. The most common compounds among the species are terpenoids, iridoids, phenolics compounds and flavonoids [4, 5]. *T. stocksianum* Boiss is a key member of the Lamiaceae family. It is used in folk medicine for the treatment of fever, sore throat, as expectorant, diabetes, foot burning sensation, body coolant and blood purifier [6, 7]. In our previous work we extracted the essential oils from *T. stocksianum* which showed excellent analgesic activity [8]. The methanolic extract, sub-fractions and crud saponins of *T. stocksianum* exhibited marked antioxidant, analgesic, cytotoxic, phytotoxic and insecticidal activities [9–11]. It has been documented that the ethyl acetate fraction of the crude methanolic extract of this plant has shown significant antidiabetic effect in Alloxan induced diabetic rabbits, which endorse the folkloric use of the plant [12]. Islam et al, explored the antiulcerogenic and cytoprotective effects of the alcoholic extract of *T. stocksianum* [13]. Radhakrishnan et al [14] determined the anti-inflammatory effect of the ethanolic crude extract of the aerial part of *T. stocksianum.* But the detail mechanism of anti-inflammatory potential is not been reported elsewhere. In order to determine the possible anti-inflammatory mechanism, crude ethanolic extract and its subsequent fractions were initially screened for anti-inflammatory activity. Finally, the most potent fraction i.e. ethyl acetate was selected for the determination of possible anti-inflammatory mechanism using various inflammatory phlogistic agents in mice.

## Methods

### Plant material

*T. stocksianum* was collected in the month of May 2012 from District Swat in the province of Khyber Pakhtunkhwa (KPK), Pakistan and was identified by Professor Dr. Nasrullah, Department of Botany, University of Malakand, Pakistan. Voucher specimen was deposited in the Herbarium of the same Department having reference number H.UOM.BG.199. Plant was washed with tape water, shad dried and pulverized to coarse powder. About 2 kg of the plant material was extracted with ethanol (80 %), yielded 7.5 % (150 g) crud extract. The ethanolic crud extract were suspended in distilled water and fractionation was carried out using successive solvent-solvent extraction method. This resulted in 26 g (1.30 %), 38 g (1.90 %), 47 g (2.35 %) and 23 g (1.15 %) of *n*-hexane, chloroform, ethyl acetate and aqueous fraction respectively.

### Experimental animal

In the present study Swiss albino mice of either sex (25-30 g) were used. Animals were procured from the Pharmacology section of National Institute of Health, Islamabad, Pakistan. Animals were maintained in suitable cages under controlled laboratory conditions of 23-25 °C with 12 h light/dark cycle and had a free access to food and water during acclimatization period. The experimental procedures were approved by the ethical committee of the Department of Pharmacy University of Malakand, KPK, Pakistan.

### Chemicals/drugs and solubility

In this study we used Carrageenan, Histamine, Chlorpheniramine maleate, Bradykinin acetate, Arachedonic acid, Caffeic acid and Prostaglandin E_2_ which were purchased from Sigma Chemicals Company, USA. While Acetylsalicylic acid used was of Reckit & Colman, Pakistan (Aspirin).

Stock solution of Bradykinin was prepared with 70 % ethanol and was further diluted with 0.1 % ethanol. Arachedonic acid was dissolved in carbonate buffer (Na_2_CO_3_, 0.2 M, pH 8.5). Caffeic acid solution was prepared in 10 % dimethylsulphoxide (DMSO). The ethanolic extract and subsequent fractions of *T. stocksianum* were prepared as 100, 200 and 300 mg in 10 ml of 10 % DMSO. Rests of the chemicals were dissolved in 0.9 % normal saline solution.

### Anti-inflammatory activity of *Teucrium stocksianum* in mice

The preliminary anti-inflammatory activity of *T. stocksianum* was evaluated on mice of either sex (25-30 g). Thirty (30) mice were divided randomly in five groups (Groups A-E) each group containing 06 mice [15]. Group A served as a negative control, received 10 ml/kg of 10 % DMSO, group B was treated with Acetylsalicylic acid 100 mg/kg (positive control), while group C, D and E received 100, 200 and 300 mg/kg, intraperitoneally, ethyl acetate fraction of *T. stocksianum* respectively. After 30 min, freshly prepared saline suspension of carrageenan (0.05 ml of 1 % w/v) was administered subcutaneously in the sub planter surface of the right hind paw of each mouse. The inflammation was immediately measured with plethysmometer (LE 7500 plan lab S.L) after injection of the irritant (carrageenan) at 1 h interval for 5 h. Paw volume of the standard drug and drug treated animals were measured at different intervals and were compared with that of negative control group animals. Percent inhibition of inflammation was calculated using the following formula;

$$ \mathrm{Percent}\kern0.5em \mathrm{inhibition}\kern0.5em =\kern0.5em \frac{\mathrm{C}\kern0.5em -\kern0.5em \mathrm{T}}{\mathrm{C}\kern0.5em \times \kern0.5em 100} $$

Where C is the average inflammation of control and T is the paw volume of tested group [16].

### Mechanism of anti-inflammatory activity of *Teucrium stocksianum* in mice

The experimental animals of either sex were randomly divided in to various groups. The animals received intraperitoneally injection of 10 % DMSO or 0.9 % saline or chlorpheniramine maleate 25 mg/kg (antihistaminic) or 100 mg/kg caffeic acid (lipoxygenase inhibitor) or aspirin 100 mg/kg or 300 mg/kg ethyl acetate fraction of *T. stocksianum.* After 30 min of the above intraperitoneal administration, paw inflammation was induced in the right hind paw of mice by sub planter injection of 0.1 ml of histamine (1 mg/ml) or arachedonic acid (0.5 % w/v) or bradykinin (20 μg/ml) or prostaglandin E2 (0.01 μg/ml). Paw volume of each mouse was immediately measured before and after the sub planter administration of different irritants (inflammatory agents) at 1, 2, 3 and 4 h.

### Statistical analysis and calculations

All the results obtained were articulated as mean ± SEM of 06 animals. One-way analysis of variance (ANOVA) followed by post hoc Dunnett’s test multiple comparison test was applied for the comparison among various groups and Student’s *t* test to determine the significance of differences between two means. Differences with *P ≤* 0.05 and lower between groups were considered significant.

Nearly all the edemogens produced the peek inflammatory response at the 3^rd^ h on the hind paw of control animal, followed by decline in swelling at 4^th^ h. Moreover, the inflammatory response evoked by Histamine, Bradykinin, Prostaglandin E_2_, Arachedonic acid and Carrageenan, were not significant in the presence of vehicles, 10 % DMSO or normal saline in mice, consequently the observations were pooled (n = 30).

## Results and Discussion

**Fig. 1:**
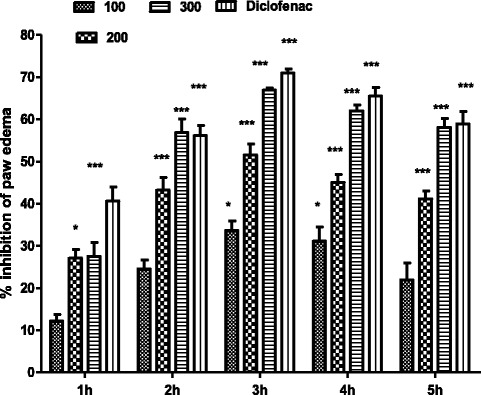
Percent inhibition produced by ethyl acetate fraction (100, 200 and 300 mg/kg) of *T. stocksianum* in carrageenan induced paw edema model in mice. Each percent point represents the mean ± SEM for group of 06 mice. Data was analyzed by ANOVA followed by post hoc Dunnett’s test. Asterisks show significant values from control. *P < 0.05, **P < 0.01, ***P < 0.001

Preliminary anti-inflammatory activity of the crude ethanolic extract and subsequent fractions of *T. stocksianum* were determined in carrageenan induced paw edema in mice. All the test samples displayed a dose dependent and significant anti-inflammatory activity. The ethyl acetate fraction (EAF) at a dose of 100, 200 and 300 mg/kg exhibited highest anti-inflammatory potential (results of rest of the fractions are not presented). EAF demonstrated excellent (66 %) anti-inflammatory action at the highest tested dose (300 mg/kg) that reached to the maximum value at 3^rd^ hour after carrageenan injection and remained significant (***P < 0.001) till 5^th^ hour of test sample administration, presented in Table 1. Aspirin has shown significant effect (70.212 %, ***P < 0.001) at a dose of 100 mg/kg at 3 h which is almost a similar effect to that produced by EAF at 300 mg/kg (Fig. 1).

**Table 1:** Concentration dependent anti-inflammatory effect of ethyl acetate fraction of *T.stocksianum* (TSEAF) in carrageenan induced paw edema test

Sample/drug	Dose mg/kg	1 h	2 h	3 h	4 h	5 h
DMSO 10 %	10 ml	0.220 ± 0.109	0.233 ± 0.08	0.235 ± 0.08	0.236 ± 0.03	0.236 ± 0.02
EAF	100	0.193 ± 0.076	0.173 ± 0.061	0.158* ± 0.05	0.163* ± 0.09	0.181 ± 0.104
	200	0.160* ± 0.051	0.13*** ± 0.051	0.116*** ± 0.05	0.130*** ± 0.04	0.135*** ± 0.07
	300	0.160* ± 0.106	0.098*** ± 0.05	0.080*** ± 0.02	0.096*** ± 0.04	0.098*** ± 0.04
Diclofenac sodium	10	0.130*** ± 0.06	0.101*** ± 0.08	0.070*** ± 0.02	0.081*** ± 0.05	0.096*** ± 0.07

Values are reported as mean ± SEM, n = 06. Data was analyzed by ANOVA followed by post hoc Dunnett’s test for multiple comparisons. Asterisks show significant values from control. *P < 0.05, **P < 0.01***P < 0.001

Carrageenan induced inflammation is a well-established non-specific protocol for the determination of anti-inflammatory action of medicinal agents. Inflammation induced by carrageenan is believed to be biphasic. The first phase is mediated by the release of serotonin and histamine while the second phase is mediated by leukotrienes and prostaglandins produced by tissue macrophages and bradykinin which peek at 3 h [17]. To ascertain the possible mechanism of inhibition, anti-inflammatory activity of EAF (300 mg/kg) was evaluated against various phlogistic agents including histamine, bradykinin, prostaglandin and arachedonic acid.

**Fig. 2:**
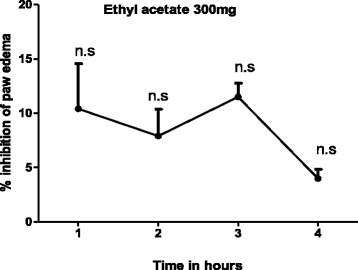
Percent inhibition produced by ethyl acetate fraction (300 mg/kg) in bradykinin induced paw edema model in mice. Each percent point represents the mean ± SEM for group of 06 mice. Data was analyzed by student’s *t* test. n.s; indicates non-significant values compared to phlogistic agent (Bradykinin)

**Fig. 3:**
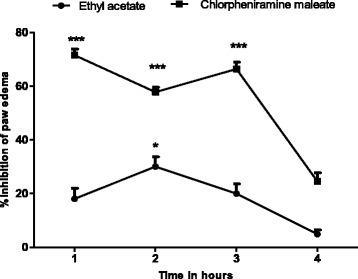
Percent inhibition produced by ethyl acetate fraction (300 mg/kg) and chlorpheniramine maleate (25 mg/kg) in histamine induced paw edema model in mice. Each percent point represents the mean ± SEM for group of 06 mice. Data was analyzed by ANOVA followed by post hoc Dunnett’s test. Asterisks show significant values from phlogistic agent (Histamine). *P < 0.05, **P < 0.01, ***P < 0.001

**Fig. 4:**
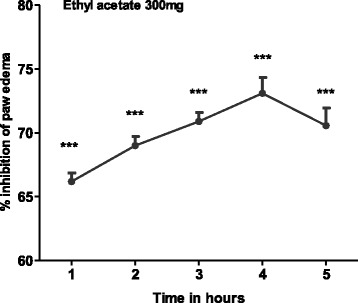
Percent inhibition produced by ethyl acetate fraction (300 mg/kg) in prostaglandin E_2_ induced paw edema model in mice. Each percent point represents the mean ± SEM for group of 06 mice. Data was analyzed by student’s *t* test. Asterisks show significant values from phlogistic agent (Prostaglandin E_2_). *P < 0.05, **P < 0.01, ***P < 0.001

**Fig. 5:**
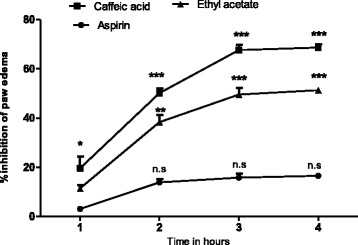
Percent inhibition produced by aspirin (100 mg/kg), ethyl acetate fraction (300 mg/kg) and caffeic acid (100 mg/kg) in arachedonic acid induced paw edema model in mice. Each percent point represents the mean ± SEM for group of 06 mice. Data was analyzed by ANOVA followed by post hoc Dunnett’s test. Asterisks show significant *P < 0.05, **P < 0.01, ***P < 0.001 and n.s; indicates non-significant values from phlogistic agent (Arachedonic acid)

The ethyl acetate fraction could not antagonize the inflammation induced by bradykinin (Table 2, Fig. 2) but a mild antihistaminic effect (18.148-31.048 %) was observed at 2 h of histamine administration, which may be due to the inhibitory effect of the extract on the release of mediators from mast cell (Table 3). Moreover, the reference standard drug chlorpheniramine maleate (antihistaminic) has significantly (65-72 %) decreased the edema induced by histamine (Fig. 3). Arachedonic acid is the main component of phospholipids of plasma membrane. Phospholipids are cleaved by an enzyme phospholipase A_2_, releases arachedonic acid which on oxidation (by cyclooxygenase) yields a strong pro-inflammatory mediator prostaglandin PGE_2_. Similarly, leukotrienes are produced as a result of arachedonic acid metabolism by lipoxygenase and could cause inflammation. This option convinced us to search out the most possible pathway of anti-inflammatory effect of *T. stocksianum* extract. In our study, we found that the plant extract significantly inhibited (66.23-73.076 %) the edematogenic effect in prostaglandin E_2_ (PGE_2_) induced paw edema (Fig. 4), signifying that the anti-inflammatory action of *T. stocksianum* extract is due to PGE_2_ inhibition. Same were the findings of Channa et al, who determined the anti-inflammatory activity of *Bacopa monniera* in rodents via prostaglandin inhibition [18]. Literature survey shows that the lipoxygenase shows significant anti-inflammatory action in carrageenan induced paw inflammation [19]. In order to determine the relative involvement of lipoxygenase pathway, edema was induced with arachedonic acid which is insensitive to cyclooxygenase inhibitors [20]. The plant extract and caffeic acid (100 mg/kg) both have significantly antagonized (51.33 %, 68.65 % at 4 h) the edematogenic effect of arachedonic acid respectively (Fig. 5), while aspirin remained unsuccessful in blocking the edema caused by arachedonic acid (Table 4). These findings suggest that the *T. stocksianum* extract possesses dual inhibitory property and has the potential to antagonize both cyclooxygenase and as well as lipoxygenase pathways of arachedonic acid metabolites, supported by the mild antihistaminic effect of the extract. Our results are in conformity with the findings of Sing et al, who reported the anti-inflammatory action in the oils extracted from *Ocimum sanctum* via dual inhibition of both pathways of arachedonic acid metabolites (cyclooxygenase and lipoxygenase) [21]. It is also well documented that calcium channel antagonists restrain the inflammation induced by various phlogistic agents like bradykinin, serotonin, prostaglandin and carrageenan [22]. Therefore, the anti-inflammatory action of *T. stocksianum* could be presumed through calcium channel blocking effect. Ali et al, have demonstrated the calcium channel blocking effect of the crude methanolic extract of *T. stocksianum* [23], which authenticated our assumption. Phytochemicals screening have confirmed the presence of flavonoids, saponins and terpenoids, all these classes of phytochemicals are responsible for multiple pharmacological actions including anti-inflammatory activity [24–26].

**Table 2:** Anti-inflammatory effect of ethyl acetate extract of *T.stocksianum,* in bradykinin and prostaglandin induced paw edema test

Treatment	Dose	1 h	2 h	3 h	4 h
Bradykinin	20 μg/ml	0.251 ± 0.09	0.220 ± 0.0093	0.198 ± 0.0105	0.210 ± 0.01125
EAF	300 mg/kg	0.225^n.s^ ± 0.012	0.201^n.s^ ± 0.054	0.175^n.s^ ± 0.08	0.201^n.s^ ± 0.0109
PGE_2_	0.01 μg/ml	0.231 ± 0.06	0.240 ± 0.057	0.251 ± 0.040	0.260 ± 0.0052
EAF	300 mg/kg	0.078*** ± 0.065	0.075*** ± 0.043	0.733*** ± 0.04	0.070*** ± 0.036

*EAF* ethyl acetate fraction, *PGE*_*2*_ prostaglandin E_2_. Values are reported as mean ± SEM, n = 06. Data was analyzed by student’s *t* test. Asterisks show significant *P < 0.05, **P < 0.01, ***P < 0.001 and n.s; indicates non-significant percent inhibition compared to respective Phlogistic agents

**Table 3:** Anti-inflammatory effect of ethyl acetate extract (EAF) of *T.stocksianum,* in histamine induced paw edema test

Test sample/drug	Dose	1 h	2 h	3 h	4 h
Histamine	1 mg/ml	0.270 ± 0.036	0.248 ± 0.06	0.231 ± 0.065	0.230 ± 0.081
CPM	25 mg/kg	0.0767*** ± 0.055	0.105*** ± 0.062	0.0783*** ± 0.075	0.183 ± 0.088
EAF	300 mg/kg	0.221 ± 0.08	0.171** ± 0.08	0.185* ± 0.088	0.218 ± 0.060

*EAF* ethyl acetate fraction. Values are reported as mean ± SEM, n = 06. Data was analyzed by ANOVA followed by post hoc Dunnett’s test. Asterisks show significant values from phlogistic agent (Histamine). *P < 0.05, **P < 0.01, ***P < 0.001

**Table 4:** Anti-inflammatory effect of ethyl acetate extract of *T.stocksianum,* in arachedonic acid induced paw edema test

Test sample/drug	Dose	1 h	2 h	3 h	4 h
Ard	0.5 % w/v	0.2050 ± 0.061	0.2300 ± 0.077	0.2516 ± 0.030	0.2500 ± 0.025
C.Acid	100 mg/kg	0.1666* ± 0.102	0.1150** ± 0.071	0.0816** ± 0.060	0.0783** ± 0.030
EAF	300 mg/kg	0.1816 ± 0.074	0.1416* ± 0.074	0.1233** ± 0.076	0.1216** ± 0.030
Aspirin	100 mg/kg	0.1983 ± 0.047	0.1983 ± 0.087	0.215 ± 0.020	0.235 ± 0.042

*Ard* arachedonic acid, *C.Acid* caffeic acid, *EAF* ethyl acetate fraction. Values are reported as mean ± SEM, n = 06. Data was analyzed by ANOVA followed by post hoc Dunnett’s test. Asterisks show significant values from Phlogestic agent (Arachedonic acid). *P < 0.01, **P < 0.001

## Conclusion

In conclusion, this research study revealed that the ethyl acetate fraction of *T. stocksianum* possesses significant anti-inflammatory activity via dual inhibition of both cyclooxygenase and lipoxygenase pathways of arachedonic acid metabolites. Moreover, highly significant effect was observed via prostaglandin inhibition. These results support the folkloric use of the plant in the treatment of various inflammatory conditions.
